# Neoadjuvant androgen deprivation therapy combined with abiraterone acetate in patients with locally advanced or metastatic prostate cancer: When to perform radical prostatectomy?

**DOI:** 10.1002/cam4.5255

**Published:** 2022-09-15

**Authors:** Ziyang Xu, Fukun Wei, Jie Wang, Sai Ma, Yi Kan, Bingheng Li, Nienie Qi, Lijun Mao

**Affiliations:** ^1^ Department of Urology The Affiliated Hospital of Xuzhou Medical University Xuzhou China

**Keywords:** cancer risk factors, neoadjuvant chemotherapy, prostate cancer, surgery

## Abstract

The surgical timing after neoadjuvant androgen‐deprivation therapy (ADT) plus abiraterone acetate (AA) for patients with locally advanced or metastatic prostate cancer (PCa) is unknown. We divided patients with locally advanced or metastatic PCa into three groups according to prostate‐specific antigen (PSA) nadir after neoadjuvant ADT plus AA: group 1 (PSA ≤ 0.2 ng/ml), group 2 (0.2 < PSA ≤ 4.0 ng/ml), and group 3 (PSA > 4.0 ng/ml).The median PSA baseline levels in groups 1, 2, 3 were 118.42 (32.03–457.78), 143.48 (17.7–8100.16), and153.35 (46.44–423.31) ng/ml, respectively. The median times of progression to CRPC in groups 1, 2,and 3 were 30, 26, and 26 months, respectively. Compared to patients with PSA nadir >0.2 ng/ml, patients with PSA nadir <0.2 ng/ml presented with longer PFS (*p* = 0.048).Our results suggested that, in patients with locally advanced or metastatic PCa, the time to progression to CRPC was longer after radical prostatectomy when PSA decreased below 0.2 ng/ml using neoadjuvant ADT plus AA.

Prostate cancer (PCa) is one of the most common malignant tumors in men. According to the global cancer statistics, 2018, PCa is the second most common cancer in the world.^1^In recent years, the incidence of PCa in China has been increasing.^2^ Although the extensive popularity of prostate‐specific antigen (PSA) screening has improved the early diagnosis rate of PCa, many patients present with locally advanced stage or distant metastasis at the time of initial diagnosis.

In recent years, an increasing number of studies have focused on the significance of radical prostatectomy in the treatment of locally advanced or metastatic PCa. In these conditions, the perioperative complication rates appear manageable, and patients may benefit from radical surgery.^3^ Research has demonstrated a decrease of the pathological stage and a lower positive surgical margin in patients receiving neoadjuvant hormonal therapy (NHT).^4^ However, NHT before radical prostatectomy did not cause a noticeable improvement in overall survival (OS) or disease‐specific survival (DSS).^5^

Compared with androgen deprivation therapy (ADT) alone, tissue androgens are maximally inhibited by ADT plus abiraterone acetate (AA). Preoperative enhancement of androgen inhibition intratumorally with ADT plus AA may reduce tumor burden.^6^ The treatment duration for traditional NHT is 3–9 months. Compared with PSA nadir >0.1 ng/ml following neoadjuvant ADT, patients with PSA nadir <0.1 ng/ml presented with better OS.^7^ The use of neoadjuvant ADT plus AA is empirical, and there is no unified conclusion on treatment duration. The surgical timing after neoadjuvant ADT plus AA for patients with locally advanced or metastatic PCa is unclear.

Based on PSA dynamics, we conducted this study to explore the surgical timing of locally advanced or metastatic PCa after neoadjuvant ADT plus AA. To the best of our knowledge, this is the first study to assess the operation timing following neoadjuvant ADT plus AA in the context of novel hormonal therapy.

We retrospectively selected 75 patients with locally advanced or metastatic PCa who underwent radical prostatectomy after neoadjuvant ADT plus AA between March 2017 and October 2019. We collected clinicopathological data including body mass index, age, Gleason score, PSA baseline level, and Eastern Cooperative Oncology Group Performance Status. In this study, we divided patients into three groups according to PSA nadir after neoadjuvant ADT plus AA: group 1 (PSA ≤ 0.2 ng/ml), group 2 (0.2 < PSA ≤ 4.0 ng/ml), and group 3 (PSA > 4.0 ng/ml). The time of progression to castration‐resistant prostate cancer (CRPC) was compared among the three groups.

CRPC was defined as castrate levels of serum testosterone <1.7 nmoL/L or 50 ng/dl, in addition to biochemical progression (three consecutive rises of PSA at least 1 week apart, leading to a 50% rises over the lowest point [with PSA > 2 ng/ml]), or radiographic progression, based on the 2020 European Association of Urology (EAU) guidelines.^8^

Tumor stage was determined according to the American Joint Committee on Cancer Tumor‐Node‐Metastasis staging system for prostate cancer (eighth edition, 2017). We ascertained the tumor grade based on the 2016 World Health Organization classification grading system, which was approved by the Institutional Review Board. Written informed consent was obtained from all patients for publication of this study.

SPSS (version 22.0; SPSS) was used for statistical analysis. Progression‐free survival (PFS) was assessed using the Kaplan–Meier survival curve analysis. PFS was defined as the time between the first diagnosis and CRPC, the last follow‐up, or death from any cause. We investigated the risk factors associated with CRPC progression by univariate and multivariate Cox regression analyses. Statistical significance was set at *p* < 0.05.

This study included 75 patients with locally advanced or metastatic PCa. The patients received neoadjuvant ADT plus AA for several months before radical prostatectomy. Of the 75 patients, 24 (32%), 31 (41.3%), and 20 (26.7%) were categorized into Group 1, Group 2, and Group 3, respectively. Table 1 shows the patients' clinical characteristics. Their median age was 71 (49–87) years. The median PSA baseline levels in groups 1, 2, 3 were 118.42 (32.03–457.78), 143.48 (17.7–8100.16), and 153.35 (46.44–423.31) ng/ml, respectively.

**TABLE 1:** Clinical features of patients in the three groups.

	<0.2 ng/ml	0.2‐4 ng/ml	>4 ng/ml	*p*
	(*n* = 24)	(*n* = 31)	(*n* = 20)	
Age (year)	72 (54–87)	73 (49–87)	70 (57–84)	0.792
ECOG performance status				0.931
0	23	29	19	
1	1	2	1	
PSA baseline level (ng/ml)				0.467
Median	118.42 (32.03–457.78)	143.48 (17.7–8100.16)	153.35 (46.44–423.31)	
Gleason score				0.726
7 points	5	7	7	
8 points	10	10	5	
9 points	7	13	8	
10 points	2	1	0	
Metastasis				0.736
No	17	22	16	
Yes	7	9	4	
Body mass index (kg/m^2^)				0.544
Median	23.13 (17.36–32.69)	23.38 (16.42–29.73)	23.66 (17.45–31.22)	

Abbreviation: ECOG, Eastern Cooperative Oncology Group.

The median follow‐up time for all patients was 27 (14–36) months. Sixty‐seven (89.3%) patients developed CRPC, and were diagnosed as progressing to CRPC through PSA changes. The median PSA nadir before radical prostatectomy of group 1, 2, and 3 were 0.093 (0.05–0.2), 0.92 (0.21–3.41), and 6.965 (4.35–9.93) ng/ml, respectively. The median times of progression to CRPC in groups 1, 2, and 3 were 30, 26, and 26 months, respectively. The PFS of the three groups is shown in Figure 1. Among the three groups,PFS showed significant differences (*p* = 0.048). Compared to patients with PSA nadir >0.2 ng/ml, patients with PSA nadir <0.2 ng/ml presented with longer PFS. Multivariate analysis showed that the PSA nadir was an independent risk factor for progression to CRPC in locally advanced or metastatic PCa (*p* = 0.036).

**FIGURE 1:**
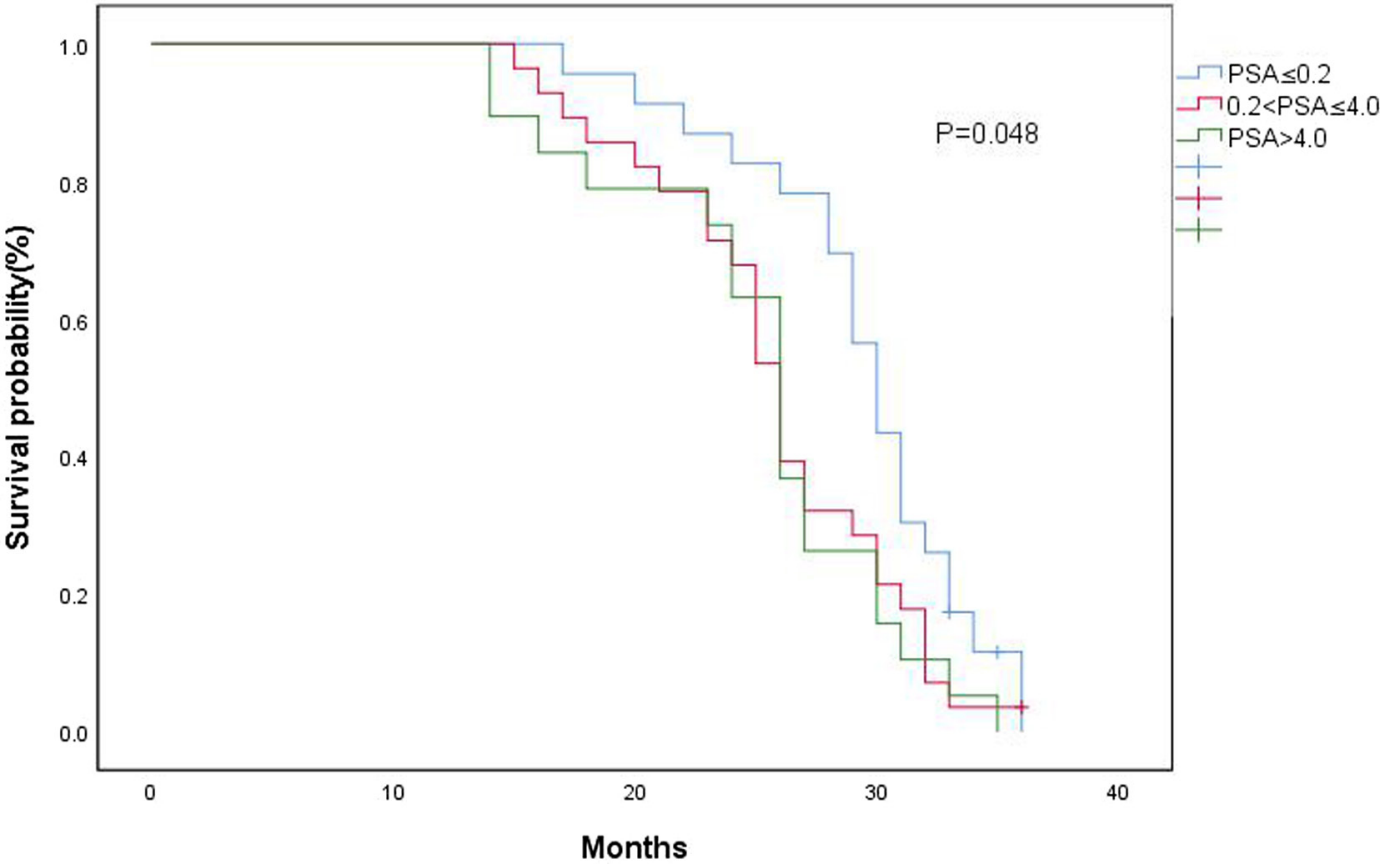
Kaplan–Meier analysis for progression‐free survival in patients with different groups. 180 × 106 mm (120 × 120 DPI).

In recent years, an increasing number of studies have focused on the significance of radical prostatectomy in locally advanced or metastatic PCa. The safety and efficacy of radical surgery in the treatment of locally advanced or metastatic PCa have been reported.^3^, ^9^, ^10^ In 2014, retrospective data from patients with metastatic PCa from the Surveillance, Epidemiology, and End Results database were used to compare 245 patients who underwent cytoreductive radical prostatectomy with 7811 patients who did not. The 5‐year OS and DSS of patients with cytoreductive surgery were 67.4% and 75.8% respectively, while those of patients without surgery were 22.5% and 48.7% respectively (*p* < 0.001).^11^ Radical prostatectomy is safe, reliable, and beneficial for locally advanced PCa. However, the treatment for locally advanced or metastatic PCa should be comprehensive and include radical surgery, and neoadjuvant, adjuvant, and systematic therapy. Gleave et al. found that 1 month of NHT can reduce serum PSA by 84%, while 3–8 months of hormonal therapy can further reduce PSA levels by 52%.^12^ Another study revealed that 3–6 months of NHT significantly reduced the positive surgical margin in patients with T3 stage disease.^13^

AA is a highly selective and irreversible CYP17 inhibitor of androgen synthesis in the prostate cancer cells, adrenal glands, and testicles, and acts by suppressing CYP17 (the key enzyme in the androgen synthesis pathway). Inhibition of CYP17 combined with ADT can inhibit maximum androgen level with complete androgen blockade.^14^ In 2014, a randomized controlled trial study explored the effect of AA combined with leuprolide acetate as neoadjuvant therapy for localized PCa.^6^ The proportion of postoperative pathological complete remission and near complete remission of patients who received neoadjuvant AA plus leuprolide acetate was significantly higher than patients who received neoadjuvant leuprolide acetate. Although the treatment duration of traditional NHT is 3–9 months, there is no unified conclusion on the medication time scheme of neoadjuvant AA plus ADT.

PSA is a serological index widely used for diagnosis, treatment evaluation, and prognostic prediction of PCa.^15^, ^16^, ^17^ Nayyar et al.^18^ reported that a higher baseline PSA level was associated with poor ADT treatment and a shorter time to progression to CRCP. Nevertheless, a systematic review demonstrated that pre‐treatment PSA levels were not related to the risk of progression or survival benefits of metastatic PCa.^19^ This is consistent with our study. The results of Southwest Oncology Group (SWOG) 9346 trial showed that the PSA nadir level after 7 months of castration was an independent predictor for the prognosis of newly diagnosed metastatic hormonal sensitive prostate cancer (mHSPC) patients. The median OS of patients with PSA nadir <0.2, 0.2–4, and >4 ng/ml was 75, 44, and 13 months, respectively (*p* < 0.0001).^20^ However, patients with metastatic PCa in the SWOG 9346 trial received ADT alone, without AA.

In this study, patients with locally advanced or metastatic PCa received neoadjuvant AA plus ADT. We found that patients with PSA nadir <0.2 ng/ml presented with longer time to progression to CRPC. The EAU guidelines pointed out that PSA levels are a prognostic factor for patients with mHSPC 7 months post‐castration.^21^ A systematic review showed that PSA nadir was prominently correlated with the survival benefit of mHSPC.^19^ Most prostate cancer cells are androgen‐sensitive prior to undergoing ADT. The decrease in serum PSA level was related to ADT. When a mass of androgen‐independent cells is present, ADT cannot significantly reduce serum PSA levels. Serum PSA levels depend on the number of androgen‐independent prostate cancer cells.^22^ Therefore, more attention should be paid to patients with a higher PSA nadir so that progression to CRPC can be identified earlier.

Our study has some limitations. The present study was a single‐center retrospective study and lacked randomization. In addition, the number of patients in the three groups was relatively small. We could not include all the factors in the multivariable model because of the small sample size. Further studies are required to confirm the optimal surgical timing after neoadjuvant AA plus ADT.

In conclusion, for patients with locally advanced or metastatic PCa after neoadjuvant AA plus ADT, when the PSA decreased below 0.2 ng/ml, the time of progression to CRPC was longer after radical prostatectomy.

## AUTHOR CONTRIBUTIONS

**Ziyang Xu:** Conceptualization (equal); methodology (equal); writing – original draft (equal). **Fukun Wei:** Conceptualization (equal); methodology (equal); writing – original draft (equal). **Jie Wang:** Conceptualization (equal); methodology (equal); writing – original draft (equal). **Sai Ma:** Data curation (equal). **Yi Kan:** Data curation (equal). **Bingheng Li:** Data curation (equal). **Nienie Qi:** Supervision (equal); writing – review and editing (equal). **lijun Mao:** Supervision (equal); writing – review and editing (equal).

## FUNDING INFORMATION

The authors received no financial support for the research, authorship, and/or publication of this article.

## CONFLICTS OF INTEREST

The authors declared no potential conflicts of interest with respect to the research, authorship, and/or publication of this article.

## ETHICS STATEMENT

The study was approved by the institutional review board from The Affiliated Hospital of Xuzhou Medical University (approval no. L20210226445).

## INFORMED CONSENT

Written informed consent was obtained from the patient for the publication of this report.

## Supporting information


Appendix S1

## Data Availability

The raw data supporting the conclusions of this article will be made available by the authors, without undue reservation.
