# Supplementary material for: Neoadjuvant androgen deprivation therapy combined with abiraterone acetate in patients with locally advanced or metastatic prostate cancer: When to perform radical prostatectomy?
Source: Cancer Med. 2022 Sep 15;12(4):4352–6. doi: 10.1002/cam4.5255 (PMC9972149; doi:10.1002/cam4.5255)
Supplement: Supplementary file 1 — Appendix S1 [file CAM4-12-4352-s001.docx]

**Supplementary Information for review and publication**

1. The results & Tables for univariate & multivariate analysis for risk factor of CRPC including neoadjuvant treatment.

Results: Risk factors in Table 1 (including age, ECOG PS, Gleason score, PSA nadir and BMI) were included in univariate and multivariate analyses. The results showed that age, ECOG PS, Gleason score and BMI were not risk factors for progression to CRPC in locally advanced or metastatic prostate cancer. Multivariate analysis showed that the PSA nadir was an independent risk factor for progression to CRPC in locally advanced or metastatic PCa (P=0.036).

Table. Multivariate analysis for progression to castration resistant prostate cancer.

|  | HR | 95%CI | P Value |
| --- | --- | --- | --- |
| Age | 0.987 | 0.955-1.021 | 0.46 |
| ECOG PS |  |  | 0.405 |
| 0 | Ref. |  |  |
| 1 | 1.580 | 0.545-4.579 | 0.399 |
| Gleason score |  |  | 0.308 |
| 7 points | Ref. |  |  |
| 8 points | 1.607 | 0.763-3.385 | 0.212 |
| 9 points | 1.437 | 0.674-3.063 | 0.348 |
| 10 points | 3.836 | 0.760-19.349 | 0.104 |
| PSA nadir |  |  | 0.036 |
| <0.2ng/ml | Ref. |  |  |
| 0.2-4ng/ml | 1.891 | 1.046-3.42 | 0.035 |
| >4ng/ml | 2.217 | 1.114-4.415 | 0.023 |
| BMI | 1.016 | 0.938-1.099 | 0.703 |

ECOG PS, Eastern Cooperative Oncology Group performance status.

Additional notes

1.Treatment plan.

These patients received neoadjuvant ADT plus AA before surgery and then underwent RP. For patients with mPC, ADT plus AA were necessary after RP. For patients with locally advanced PC, when the PSA was found detectable or persistent after RP, we would arrange adjuvant RT after the urinary continence was recovered. For patients with lymph node metastasis, adjuvant ADT was necessary after surgery.

2.Criteria of neoadjuvant treatment & continuation of the treatment after surgery.

Prostate cancer patients with stage T3/4N0M0 or lymph node positive (TxN1M0), or metastatic (TxNxM1) were include in this study.

These patients received neoadjuvant ADT plus AA before surgery and then underwent RP. For patients with mPC, ADT plus AA were necessary after RP. For patients with locally advanced PC, when the PSA was found detectable or persistent after RP, we would arrange adjuvant RT after the urinary continence was recovered. For patients with lymph node metastasis, adjuvant ADT was necessary after surgery.

3.Pathological result and nadir of PSA after surgery.

The number of patients with tumor downstaging of the three groups after surgery were 15,11and 4, respectively.

The number of patients with pathologic complete response (PCR) of the three groups after surgery were 7,3,and 1,respectively.

The median PSA nadir of the three groups after surgery were 0.02, 0.14, and 0.52 ng/ml, respectively.
